# Actinomycosis (Lumpy Jaw)

**Published:** 1892-02

**Authors:** R. W. Hickman


### ACTINOMYCOSIS.

### LUMPY JAW.

By R. W. Hickman, V. M. D.

In the noted trial known as the Peoria Lumpy Jaw case, *Greenhut vs. Pearson*, if no other benefit were gained than that of creating a new and intense interest in this disease, and more particularly in the nature and life history of the actinomyces bovis, the gain, it appears to me, will be of almost inestimable value, both to the stockmen and to the veterinary profession.

As actinomycosis is comparatively seldom met with in the East, I never gave the disease any especial attention until the organization of the export cattle inspection work at the Union Stockyards of Chicago, and then only from the diagnostic standpoint. I, however, realized its importance, and also an inclination to study the disease more closely ; and as we have had no English literature of value on the subject until very recently, I was obliged to content myself with finding the simple ray fungus, which is always present, and by this time familiar to every student of veterinary medicine.

It will be entirely unnecessary to go into a detailed description of the disease itself, that is, of the tumor, its location, appearance, etc., as all are at least familiar with this. I shall, therefore, confine myself to a description of the germ or actinomyces, following with some comments upon the inspection that has led to a closer investigation of this trouble. The actinomyces bovis is best described as a schizomyzetes, in which the cladothrix formation occurs. To simplify the matter we will commence with the starting point, which is the cocci, and endeavor to give a simple description of their growth and muliplication. These cocci multiply by elongation and subsequent fission. When excretory material is carried away and proper nutrition is available, and surroundings are favorable to their growth, they tend to assume the leptothrix or cladothrix form ; or when undisturbed by the surrounding leucocytes their growth and multiplication may be after the manner of the streptococcus. They may frequently, however, be found irregularly grouped together in staphylococcus heaps. Some of the cocci, by elongation, give rise directly to short bacillary forms and through these to long filaments. These may, through fission, multiply and form new cocci, and then new colonies, or for the want of the elements conducive to their growth and development, instead of the cladothrix formation and subsequent fission, they may have a swelling of the end of the leptothrix thread, producing the club-like form and the loss of their power of multiplication, or by the failure of the necessary supply of pabulum, or again by the effect of the accumulation of their own excretory material, which is toxic to them. Under either of these last circumstances a large majority of the threads tend to develop clubs at their outer ends, and the clubs are involution forms ; and thus the central cocci and the remainder of the filaments disintegrate, leaving the clubs, which offer a greater passive resistance to the surrounding leucocytes, and may persist for an indefinite period.

Actinomycosis is the result of the activity of a living organism introduced into and existing as a parasite in the animal tissues. It is epiphytic in its nature, living on the outer surfaces of barley and other cereals. The club-shaped organism is really an involution stage, although it was, for a long time, thought the cause of the disease, until repeated attempts at cultivation proved, through utter failure in the results, that investigators had been laboring under a misapprehension. The characteristic growth is the mycelial thread-like mass which appears to develop from the cocci. While the clubs, or what has been the characteristic diagnostic element, instead of being spore bearers are merely encysted or thick-walled involution forms.

For the benefit of those who are not so fortunate as to be possessed of or have access to a microscope, and are obliged to depend largely upon descriptions of micro-organisms and their effects, I will repeat, and at the same time endeavor to explain, the nature and form of these infinitesimal organisms with the excessively long names. We will take the old illustration of the actinomyces, namely, the field daisy. Now, we want to bear in mind that a colony consists of three distinct elements : first, the cocci, the smallest of all forms, or bacteria. Under the name bacteria may be included all of these simple-rounded, rod-shaped, thread-like forms of vegetable organisms beloeging to the class of lower fungi, and designated by the Germans as schizomycetes ; a name applied because of the process by which they divide transvers to the longitudinal axis in the case of the rod-like forms, but varying somewhat in the cladothrix form.

If micro-cocci increase in length to rods two and one-half times their diameter, they may be designated bacillary forms.

Bacilli increase in length and become more or less jointed as vegetative division takes place, forming long, delicate jointed threads, spoken of as leptothrix forms when there is no branching, but if pseudo ramifications are present, such as are found in the cladothrix dichotoma, and are due to vertical division taking place in one of the terminal cells, we have the cladothrix form. Upon the examination of the fungus under a high power microscope, the specimen being properly stained (we use almost exclusively Gram's method) the picture presented by the organism will be like the head of a daisy, the sterile flowers in the centre corresponding to the club shape rays ; and if we imagine two of these heads placed closely together, stem to stem, or base to base, we have a pretty accurate idea of the appearance of the ray fungus as a whole. Now the organism in sections will present the appearance of sections through the little ball formed by placing the two heads together as above described. Prof. McFadyean observed, "The first element is a coccus. These cocci are usually arranged in chains, consisting of ten or fifteen elements. A few of them are frequently found in the centre of the mass, but immediately outside this they are exceedingly numerous, so numerous, indeed, that they appear to be more like masses than chains. As they approach the margin again, the chains radiate outwards again, and are distinctly seen. Where they are not very numerous, large cocci may be seen undergoing vegetative division, diplococci. In some slides mounted during the second week in January, 1892, by Dr. Norgaard in our office, all of these conditions were beautifully shown. The elements, with the exception of the larger clubs, take the violet stain beautifully ; the tumor tissues and club forms are in different shades of light brown to yellow. Leucocytes may also be seen of a yellowish light brown, containing a large number of cocci stained a violet. The second element is the leptothrix or cladothrix form. This in one of our sliders presented a beautiful picture, there being a number of them lying in various directions, interlacing one another and forming a veritable network in violet color. The third element, the club form, although seeming to bear a close relation to the leptothrix form just mentioned, appear to have lost the interest formerly felt in them, except from the diagnostic standpoint, because of the frequent and constant failure of experimenters in their attempts to make cultures from them ; but as a diagnostic characteristic, they will be of leading importance, because of their constancy, in the most indifferently mounted specimen for diagnostic purposes. And to the presence of these, too, is largely due the beauty of the picture and the daisy formation, the larger clubs representing in a striking degree the petals of the flower.

In relation to these clubs, it will be noticed that the small new formed ones take the violet stain, but there appears after a while an envelope loosely surrounding these violet clubs, resulting from a process simulating an œdema, only that the fluid is absorbed from without, which increases the size of the membrane or sort of cellulose covering, and the original club in many instances is now broken up, forming large clusters or heaps of violet micro-cocci forms, sometimes toward either end or in the middle of the envelope, as if suspended by the transparent jelly-like contents of the yellowish brown membrane.

It will probably be interesting to veterinarians to know that some of the lesions of actinomycosis and tuberculosis so closely simulate each other that it is almost, if not quite, impossible to differentiate the one from the other without the aid of the microscope.

The radical views held by some, that all cattle showing the least evidence of actinomycosis should be condemned and consigned to the rendering tank without regard either to the condition of the animal or the progress the disease has made, is an unnecessarily severe measure, and in many cases an injustice to the owner. In Denmark, where the disease is probably as well understood as in any country in the world, so little is thought of the possibility of one actinomycotic animal communicating the disease to others of the herd that the diseased animal is not separated from them until the tumor commences to suppurate; then the animal is slaughtered.

The fact that actinomycosis can be conveyed by inoculation admits of the possibility of accidental inoculation—that is, by the discharge from the actinomycotic tumor coming in contact with an abraided surface, and the disease resulting therefrom. It is generally considered by investigators, and we may add a fairly well established fact, that the actinomyces live an epiphytic life on barley and other cereals, and may be introduced into the animal organism from without through wounds or abrasions of the skin or alimentary canal. In evidence we have cases too numerous to mention, but never a single case has been traced to the consumption as food of the meat of the animal affected with actinomycosis.

German Sims Woodhead, in his Contemporary Science, Series Backteria and their Products, says as to the mode of infection: " It has been pointed out that the actinomyces has been found lodged in the crypts of the tonsils of the pig and of the human subject; that it may be introduced from without through wounds, as in cases of scirrhous cord in horses, or through inoculation by means of accidental scratches of the skin or of the mouth and pharynx. And it is recorded that a case of primary media-stinal actinomycosis in a human subject was in one case supposed to be traced to the perforation of the back of the throat by a barley spikelet swallowed by the patient. In pigs the mammary affection is thought to be due to the entrance of the actinomyces into the teat ducts. Actinomycosis shows no tendency to localize in the flesh, and not a single instance has been given where the tumor has appeared in the muscles except at the primary point of infection, so that all evidence points to the conclusion that man and the domesticated animals receive the infection from the same source and in the same ways."

It has been my privilege (through the desire manifested by the Hon. Secretary of Agriculture and the Chief of the Bureau of Animal Industry to offer every facility for the advancement of veterinary science in the Department, to have placed at my disposal a microtome, a Zeiss microscope, an apochromatic emersion lense and Dr. Victor A. Norgaard assigned to special microscopic work here) to see all and study in part this very interesting micro-organism in its various forms. My work being largely of an executive character, the time would not otherwise have been afforded me. We are now arranging to make some cultures, which will be followed by some inoculations, and hope to be able at some future time to interest the readers of the JOURNAL with a report of our further investigation.

To be on the safe side I would reject or condemn any and all animals suffering from any disease or condition that could possibly render their flesh injurious for human food. This though includes numerous conditions other than lumps on the jaw, face or neck. Animals suffering from supperating actinomycotic or other tumors should be condemned on general grounds, and also on scientific principles, and not simply for sentimental reasons. Instead of this sentimentalism and furor about the one disease in question, as if it were the only one in the category to which the bovines are subject, why not put into operation the practical common sense inspection of milk, the condition, healthfulness and hygienic surroundings of the cows which furnish the supply ? Do we not all know that here lies by far the greatest danger, not only through tubercular and actinomycotic deposits in the udder, but various other diseases and conditions liable to exist, especially among the cows that furnish a large proportion of the milk to families in our large cities? Many of these cows are confined in close, poorly ventilated stables or sheds and kept in a dirty condition that is not in any way conducive to their health. I trust the time will soon come when the necessary measures shall be adopted, and a sufficiently liberal appropriation made to include in the cattle inspection system of this country a careful inspection of milch and dairy cows by a competent veterinarian, both as to the healthfulness of the animals supplying the milk consumed by our people, and as to the hygienic conditions surrounding them.

I do not feel that I can close this article without a reference to the United States Bureau of Animal Industry, which, notwithstanding the uncharitable comments that have been made by some malcontents and soreheads, has, under the direction of our competent and estimable chief, Dr. D. E. Salmon, done more for the veterinary profession in this country in a scientific way and otherwise than any other institution in our land. It may be asserted by these same individuals, who possibly long ago abandoned the hope of rising to positions of prominence and respectability in the profession and are determined to gain a vulgar notoriety which perfectly satisfies certain grades of minds, that I am prejudiced in favor of the Department, having been an employee for nearly four years. Just here I will say this is another proof that the Bureau is not being run in the interests of politicians. It will be four years next March since I was appointed, which appointment followed my own individual application to the present chief, who has ever been casting his eye over the country looking for capable veterinarians, who would honestly and earnestly serve the interests of the Bureau, and at the same time enjoy the confidence and respect of the people with whom they came in contact. I wish to say that my services have been recognized just as they would have been by any good institution or mercantile firm without any political influence on my part whatever.

It has been my privilege to serve the Department in an official capacity in my own State, Pennsylvania, and subsequently for more than two years in New York State, and now a little over one year in the State of Illinois, and I can unhesitatingly say that at Chicago and elsewhere the inspection conducted under the United States Bureau of Animal Industry has and will be honestly and conscientiously prosecuted. The inspectors having no selfish interests to serve are perfectly aware in what neglect of duty, dishonesty or incompetency would result. I do not wish that it should be inferred from this that it is never found necessary to make changes in the Department, because of the discovery of the above mentioned faults in an occasional individual. No matter what the nature of the business may be its success must depend largely upon the character of its employees, consequently there can be but one object in the selection of men for this work, and that is to procure the qualifications here alluded to.

It was my intention when I sat down to confine myself entirely to the subject in hand, but my feelings have been stirred and injured by the contemptible articles that have appeared in our representative journals, assailing those whom I know through close business association to be (I want to emphasize this) in every way competent, and in honor above suspicion or reproach.

